# Effects of Atrial Fibrillation Screening According to N-Terminal Pro-B-Type Natriuretic Peptide: A Secondary Analysis of the Randomized LOOP Study

**DOI:** 10.1161/CIRCULATIONAHA.123.064361

**Published:** 2023-04-15

**Authors:** Lucas Yixi Xing, Søren Zöga Diederichsen, Søren Højberg, Derk W. Krieger, Claus Graff, Ruth Frikke-Schmidt, Morten S. Olesen, Axel Brandes, Lars Køber, Ketil Jørgen Haugan, Jesper Hastrup Svendsen

**Affiliations:** 1 Departments of Cardiology (L.Y.X., S.Z.D., M.S.O., L.K., J.H.S.), Copenhagen University Hospital–Rigshospitalet, Denmark.; 2 Clinical Biochemistry (R.F.-S.), Copenhagen University Hospital–Rigshospitalet, Denmark.; 3 Department of Cardiology, Zealand University Hospital–Roskilde, Denmark (L.Y.X., K.J.H.).; 4 Department of Cardiology, Copenhagen University Hospital–Bispebjerg, Denmark (S.Z.D., S.H.).; 5 Department of Neurology, Mediclinic City Hospital, Dubai, United Arabic Emirates (D.W.K.).; 6 Department of Neuroscience, Mohammed Bin Rashid University of Medicine and Health Science, Dubai, United Arabic Emirates (D.W.K.).; 7 Department of Health Science and Technology, Aalborg University, Denmark (C.G.).; 8 Department of Clinical Medicine (R.F.-S., L.K., J.H.S.), Faculty of Health and Medical Sciences, University of Copenhagen, Denmark.; 9 Biomedical Sciences (M.S.O.), Faculty of Health and Medical Sciences, University of Copenhagen, Denmark.; 10 Department of Clinical Research, Faculty of Health Sciences, University of Southern Denmark (A.B.).; 11 Department of Cardiology, Esbjerg Hospital – University Hospital of Southern Denmark (A.B.).; 12 Department of Cardiology, Odense University Hospital, Denmark (A.B.).

**Keywords:** atrial fibrillation, mass screening, natriuretic peptide, brain, stroke

## Abstract

Research suggests NT-proBNP (N-terminal pro-B-type natriuretic peptide) to be a strong predictor of incident atrial fibrillation (AF) and stroke. However, its utility in AF screening remains unknown. The aim of this study was to investigate NT-proBNP as a potential marker for screening efficacy with respect to AF yield and stroke prevention.

**Methods:**

In the LOOP Study (Atrial Fibrillation Detected by Continuous ECG Monitoring Using Implantable Loop Recorder to Prevent Stroke in High-Risk Individuals), 6004 AF-naïve individuals at least 70 years old and with additional stroke risk factors were randomized 1:3 to either screening with an implantable loop recorder (ILR) and initiation of anticoagulation upon detection of AF episodes lasting ≥6 minutes or usual care (control). This post hoc analysis included study participants with available NT-proBNP measurement at baseline.

**Results:**

A total of 5819 participants (96.9% of the trial population) were included. The mean age was 74.7 years (SD, 4.1 years) and 47.5% were female. The median NT-proBNP level was 15 pmol/L (interquartile range, 9–28 pmol/L) corresponding to 125 pg/mL (interquartile range, 76–233 pg/mL). NT-proBNP above median was associated with an increased risk of AF diagnosis both in the ILR group (hazard ratio, 1.84 [95% CI, 1.51–2.25]) and the control group (hazard ratio, 2.79 [95% CI, 2.30–3.40]). Participants with NT-proBNP above the median were also at higher risk of clinical events compared with those having lower levels (hazard ratio, 1.21 [95% CI, 0.96–1.54] for stroke or systemic embolism [SE], 1.60 [95% CI, 1.32–1.95] for stroke/SE/cardiovascular death, and 1.91 [95% CI, 1.61–2.26] for all-cause death). Compared with usual care, ILR screening was associated with significant reductions in stroke/SE and stroke/SE/cardiovascular death among participants with NT-proBNP above median (hazard ratio, 0.60 [95% CI, 0.40–0.90] and 0.70 [95% CI, 0.53–0.94], respectively) but not among those with lower levels (*P*_interaction_=0.029 for stroke/SE and 0.045 for stroke/SE/cardiovascular death). No risk reduction in all-cause death was observed in either NT-proBNP subgroup for ILR versus control (*P*_interaction_=0.68). Analyzing NT-proBNP as a continuous variable yielded similar findings.

**Conclusions:**

In an older population with additional stroke risk factors, ILR screening for AF was associated with a significant reduction in stroke risk among individuals with higher NT-proBNP levels but not among those with lower levels. These findings should be considered hypothesis generating and warrant further study before clinical implementation.

**Registration:**

URL: https://www.clinicaltrials.gov; Unique identifier: NCT02036450.

**Clinical Perspective**

**What Is New?**

- In an older population with additional stroke risk factors but without known atrial fibrillation (AF), a higher NT-proBNP (N-terminal pro-B-type natriuretic peptide) level at baseline was associated with increased risk of AF, stroke, and death.
- The yield of AF screening using an implantable loop recorder compared with usual care was higher among participants with lower NT-proBNP levels than those with higher levels.
- Implantable loop recorder screening was associated with a significantly reduced stroke risk among participants with higher NT-proBNP levels but not among those with lower levels.

**What Are the Clinical Implications?**

- Higher NT-proBNP levels may identify individuals who are more likely to benefit from AF screening.
- Using NT-proBNP for risk stratification may improve the cost-effectiveness of AF screening.

It is well known that atrial fibrillation (AF) contributes to a substantially increased risk of stroke.^1,2^ As the majority of AF episodes remain asymptomatic,^2,3^ the timely diagnosis of AF seems to be a major challenge in clinical practice. The LOOP Study (Atrial Fibrillation detected by Continuous ECG Monitoring Using Implantable Loop Recorder to Prevent Stroke in High-Risk Individuals; NCT02036450) was a randomized trial to assess systematic AF screening with implantable loop recorder (ILR) in an older population with high stroke risk based on the CHA_2_DS_2_-VASc score (congestive heart failure, hypertension, age ≥75 years [2 points], diabetes, stroke/thromboembolism [2 points], vascular disease, age 65-74 years, sex category [female]). However, the study reported only a nonsignificant reduction in stroke,^4^ suggesting that not all subclinical AF is worth screening for. Further insights into risk stratification of subclinical AF are therefore warranted to refine future screening strategies.

NT-proBNP (N-terminal pro-B-type natriuretic peptide) is a cardiac biomarker that has been linked to incident AF and stroke in previous research.^5–11^ Data from several randomized trials also showed that adding NT-proBNP to the CHA_2_DS_2_-VASc score significantly improved the prediction of stroke in patients with AF.^9–11^ However, data on the relationship between NT-proBNP and effects of AF screening are lacking. To fill this knowledge gap, we conducted a secondary analysis of the LOOP Study to examine the effects of ILR screening compared with usual care on stroke prevention according to NT-proBNP.

## Methods

The study data underlying this article cannot be shared publicly for ethical reasons, but the methodology will be shared on reasonable request to the corresponding author (J.H.S.), who has full access to all data in the study and takes responsibility for data integrity and analysis.

### The LOOP Study

The LOOP Study was a randomized, controlled, multicenter trial that investigated long-term continuous AF screening in individuals aged 70 to 90 years and with ≥1 additional stroke risk factor (arterial hypertension, diabetes mellitus, heart failure, or previous stroke). A total of 6004 participants were enrolled at 4 centers in Denmark (from January 31, 2014, to May 17, 2016) and randomized to either ILR screening or usual care (the control group) in a 1:3 ratio. During ILR monitoring, oral anticoagulation (OAC) was recommended when any new-onset ILR-detected AF episode ≥6 minutes was confirmed by ≥2 senior cardiologists. The trial design and the primary reporting of the LOOP Study have been published previously.^4,12^

The LOOP Study was approved by the Regional Scientific Ethics Committee for the Capital Region of Denmark (H-4-2013-025) and conducted in accordance with the Declaration of Helsinki. Oral and written informed consent was obtained from all study participants at inclusion.

### NT-proBNP Levels

In the LOOP Study, whole-blood samples were taken from study participants at the local centers upon randomization. NT-proBNP levels were measured in 2 central hospital laboratories with the sandwich electrochemiluminescence immunoassay (Roche Diagnostics GmbH, Germany) and the Cobas 8000 analyzer system (Roche Diagnostics) in accordance with manufacturer instructions.

In the present study, we included the participants with available NT-proBNP measurement at baseline and further divided them into 2 subgroups based on the median NT-proBNP level, which was also identical to the clinically reasonable cutoff for AF screening, as previously proposed and currently used in ongoing trials.^13,14^ Supplementary analyses were further conducted to assess NT-proBNP as a continuous variable. NT-proBNP levels are reported as picomole per liter (pmol/L). The conversion factor for NT-proBNP expressed as picogram per milliliter (pg/mL) is 1 pg/mL=0.12 pmol/L (Table S1).^15^

### Study Outcomes and Follow-Up

All participants in the LOOP Study were followed up from randomization until death or January 28, 2021, with 0 participants lost to follow-up. As in primary reporting of the LOOP Study, the primary outcome for this secondary analysis was a composite end point of stroke or systemic embolism (SE). Secondary outcomes were the composite end point of stroke, SE, or cardiovascular death and the end point of all-cause death. Other outcomes of interest included diagnosis of AF, ILR-detected AF episode ≥24 hours, and OAC initiation. The primary and secondary outcomes were adjudicated by a clinical end point committee blinded to randomization assignment in accordance with prespecified criteria,^12^ whereas the end point of AF episode ≥24 hours was evaluated by ≥1 experienced physician.

### Statistical Analysis

Baseline characteristics are summarized as frequency with percentage for categorical variables, and the distributions were compared with the χ^2^ test. For continuous variables, the characteristics are summarized as mean with SD or median with interquartile range when appropriate and further compared using *t* test and Kruskal-Wallis test, respectively.

All outcomes were analyzed as time to first event. Crude event rates (expressed as events per 100 person-years) were calculated with Poisson regression, whereas cumulative incidences were determined with the Kaplan-Meier estimator for all-cause death and with the Aalen-Johansen estimator with death as a competing risk for all other outcomes. The relative risks of outcomes between NT-proBNP subgroups were estimated in the entire study cohort with multivariable cause-specific Cox regression models adjusted for sex, age, body mass index, weekly alcohol consumption, smoking pack-years, estimated glomerular filtration rate, total cholesterol concentration, hypertension, diabetes, previous stroke, heart failure, valvular heart disease, ischemic heart disease, and peripheral artery disease. For primary and secondary outcomes, NT-proBNP was also analyzed as a continuous variable with restricted cubic spline regression (knots located at the 5th, 35th, 65th, and 95th percentiles) in the multivariable Cox models.

The relative risks of study outcomes for ILR screening compared with usual care were examined according to NT-proBNP subgroups in cause-specific Cox regression models, with the potential interaction tested by further adding an interaction term to the models. The intention-to-treat principle was applied in the analyses of screening effects. In addition, linear and spline-based models were used to assess the associations between continuous NT-proBNP and the relative risks of primary and secondary outcomes for ILR versus control, for which we employed the Akaike information criterion to find the model with an optimal balance between fit and parsimony.^16,17^ Finally, a model assuming a linear relationship between log-transformed NT-proBNP and log hazards of primary and secondary outcomes in each randomization group was selected due to the lowest Akaike information criterion score (Table S2). Furthermore, as risk factor management is an important part of guideline-directed AF treatment and high systolic blood pressure (SBP) in particular constitutes a predominant stroke risk factor,^2,18,19^ it was explored by determining blood pressure changes over the first 3 years of follow-up according to randomization assignment in each NT-proBNP subgroup. The analysis was performed using a constrained linear mixed model with an unstructured covariance pattern to account for repeated measurements in the same subject.

The Cox proportional hazard assumption was tested with scaled Schoenfeld residuals, and any variables violating this were treated with stratification to allow different baseline hazards. All data analyses were performed with R (version 4.1.0, R Core Team). Two-sided values of *P*≤0.05 set the statistical significance.

## Results

Of the 6004 participants enrolled in the LOOP Study, 5819 (96.9%) had available NT-proBNP measurement at baseline and were included in this secondary analysis, with a mean age of 74.7 years (SD, 4.1 years) and with 47.5% being female. The median level of NT-proBNP at baseline was 15 pmol/L (interquartile range, 9–28 pmol/L) corresponding to 125 pg/mL (IQR, 76–233 pg/mL) (Figure S1). The Table summarizes the baseline characteristics according to NT-proBNP subgroups. Participants with NT-proBNP >15 pmol/L were older, were more likely female, and had higher SBP but lower body mass index compared with those having lower NT-proBNP levels. Cardiovascular comorbidities such as heart failure, previous stroke, ischemic heart disease, valvular heart disease, and peripheral artery disease were also more prevalent among participants with NT-proBNP above median.

**Table.**
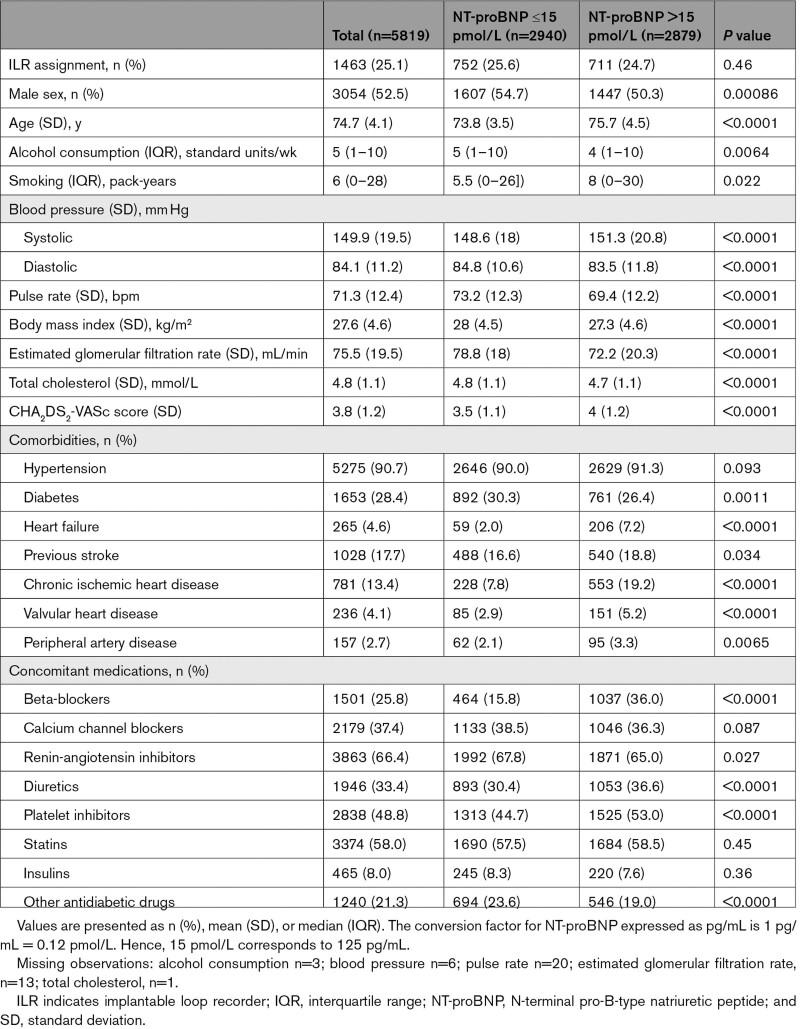
Baseline Characteristics According to NT-proBNP Subgroups

### Primary and Secondary Outcomes According to NT-proBNP

**Figure 1.**
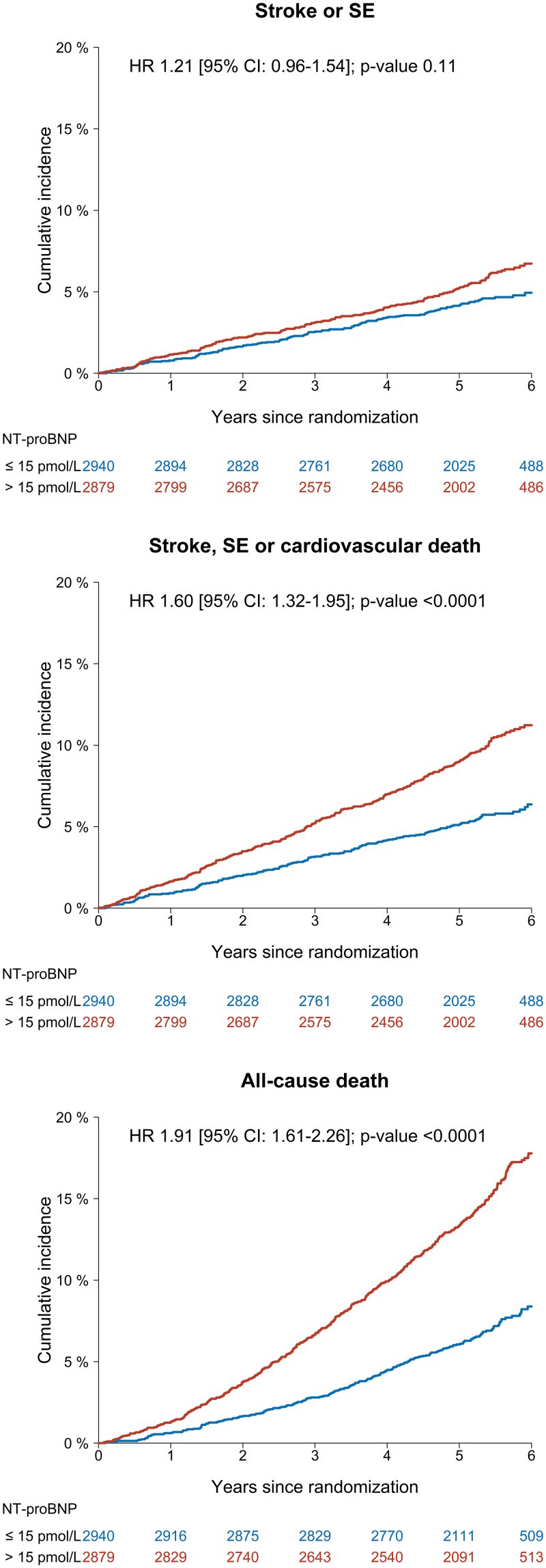
**Cumulative incidences of primary and secondary outcomes according to NT-proBNP subgroups.** Absolute risks of stroke/systemic embolism (SE), stroke/SE/cardiovascular death, and all-cause death in the entire study cohort according to NT-proBNP (N-terminal pro-B-type natriuretic peptide) subgroups. Cumulative incidences were plotted with the Kaplan-Meier estimator for all-cause death and the Aalen-Johansen estimator for other outcomes with death as competing risk. Hazard ratios (HRs) with 95% CI and *P* values were determined in cause-specific Cox models adjusted for sex, age, body mass index, weekly alcohol consumption, smoking pack-years, estimated glomerular filtration rate, total cholesterol concentration, hypertension, diabetes, previous stroke, heart failure, valvular heart disease, ischemic heart disease, and peripheral artery disease. The conversion factor for NT-proBNP expressed as pg/mL is 1 pg/mL=0.12 pmol/L. Hence, 15 pmol/L corresponds to 125 pg/mL.

Among 5819 participants included, 310 had stroke/SE (307 stroke and 3 SE), and 669 died during a median follow-up duration of 5.4 years (interquartile range, 4.9–5.8 years). For the entire study cohort, the event rate of the primary outcome of stroke/SE was numerically higher among participants with NT-proBNP >15 pmol/L (1.21 [95% CI, 1.04–1.40] per 100 person-years) than those with lower levels (0.89 [95% CI, 0.74–1.05] per 100 person-years), as indicated by a hazard ratio (HR) of 1.21 (95% CI, 0.96–1.54). For secondary outcomes, participants with NT-proBNP >15 pmol/L were at significantly increased risks of both stroke/SE/cardiovascular death and all-cause death compared with those having lower levels (HR 1.60 [95% CI, 1.32–1.95] and 1.91 [95% CI, 1.61–2.26], respectively). The cumulative incidences and crude event rates of primary and secondary outcomes according to NT-proBNP subgroups are presented in Figure 1 and Table S3. When assessed as a continuous variable, elevating NT-proBNP levels were significantly associated with increasing risks of stroke/SE, stroke/SE/cardiovascular death, and all-cause death (*P*=0.0026, <0.0001, and <0.0001, respectively; Figure S2).

### ILR Screening Effects on Primary and Secondary Outcomes According to NT-proBNP

**Figure 2.**
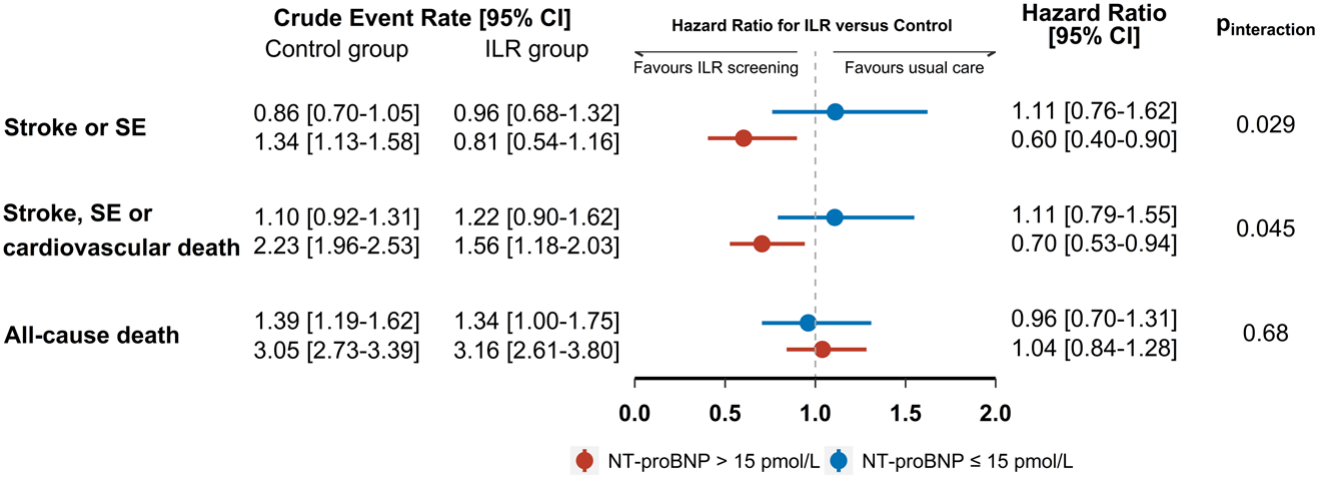
**ILR screening effects on primary and secondary outcomes according to NT-proBNP subgroups.** Event rates and hazard ratios of stroke/systemic embolism (SE), stroke/SE/cardiovascular death, and all-cause death for implantable loop recorder (ILR) screening vs usual care according to NT-proBNP (N-terminal pro-B-type natriuretic peptide) subgroups. Crude event rates are presented as number of events per 100 person-years with 95% CI and were estimated with Poisson regression. Hazard ratios and *P* values for interaction were determined in cause-specific Cox models. The conversion factor for NT-proBNP expressed as pg/mL is 1 pg/mL=0.12 pmol/L. Hence, 15 pmol/L corresponds to 125 pg/mL.

**Figure 3.**
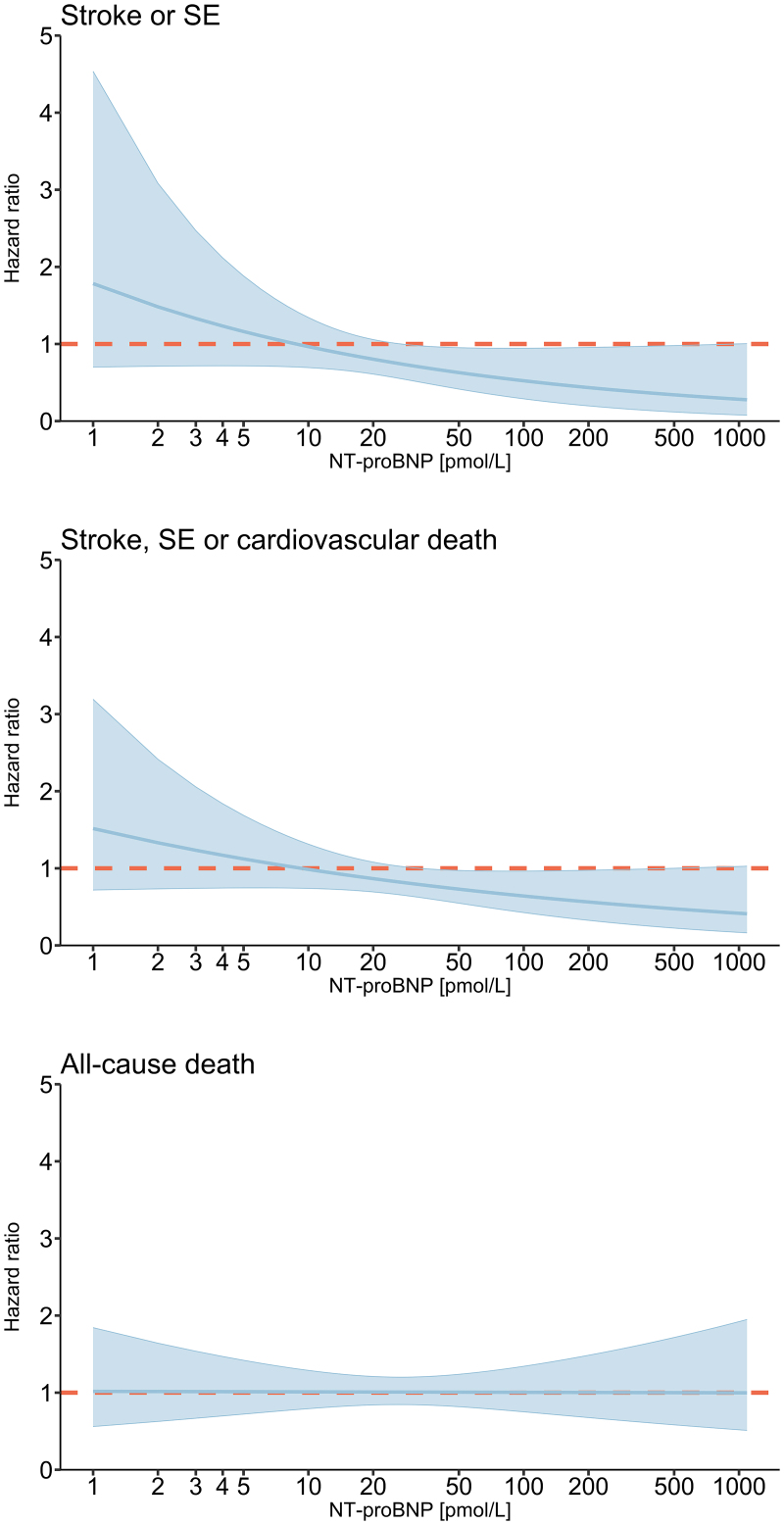
**Associations of ILR screening effects on primary and secondary outcomes with NT-proBNP as a continuous variable.** Effects of implantable loop recorder (ILR) screening vs usual care on stroke/systemic embolism (SE), stroke/SE/cardiovascular death, and all-cause death according to NT-proBNP (N-terminal pro-B-type natriuretic peptide) as a continuous variable. Hazard ratios were estimated in cause-specific Cox models, with the control group as reference. Colored areas represent 95% CI. The conversion factor for NT-proBNP expressed as pg/mL is 1 pg/mL=0.12 pmol/L.

Figure 2 illustrates the relative risks of primary and secondary outcomes for ILR screening compared with usual care according to NT-proBNP subgroups. The risk of stroke/SE was not statistically different across the randomization groups among participants with NT-proBNP ≤15 pmol/L at baseline (HR 1.11 [95% CI, 0.76–1.62]), whereas a significant risk reduction by ILR screening compared with usual care was observed among participants having higher NT-proBNP levels (HR 0.60 [95% CI, 0.40–0.90]; *P*_interaction_=0.029). The 6-year cumulative incidence was estimated to be 4.28% (95% CI, 2.71–5.86) in the ILR group and 7.51% (95% CI, 6.26–8.76) in the control group among participants with NT-proBNP >15 pmol/L (Figure S3), corresponding to an absolute risk reduction of 3.23% and a number needed to screen to avoid one stroke/SE of 31 (ie, 100/3.23). Likewise, ILR screening was associated with a significant risk reduction of stroke/SE/cardiovascular death only among participants with NT-proBNP >15 pmol/L (HR 0.70 [95% CI, 0.53–0.94]) but not among those with lower levels (HR 1.11 [95% CI, 0.79–1.55]; *P*_interaction_=0.045). For all-cause death, no significant risk difference between the randomization groups was found in either NT-proBNP subgroup. With NT-proBNP analyzed as a continuous variable, similar effect patterns were observed on stroke/SE (*P*_interaction_=0.084), stroke/SE/cardiovascular death (*P*_interaction_=0.11), and all-cause death (*P*_interaction_=0.98); Figure 3. Both for stroke/SE and stroke/SE/cardiovascular death, the risk reduction for ILR screening compared with usual care appeared to increase with higher NT-proBNP levels, and the trend curves crossed the reference line (HR = 1) at an NT-proBNP level of 9 pmol/L. With further exploration using NT-proBNP cutoffs corresponding to the 75th (ie, >28 pmol/L) and 95th (ie, >76 pmol/L) percentiles, the absolute risk differences in stroke/SE were estimated to be 3.26% (6-year cumulative incidence, 8.61% [95% CI, 6.77–10.45] for control versus 5.35% [95% CI, 2.83–7.88] for ILR) and 4.68% (6-year cumulative incidence, 11.55% [95% CI, 7.04–16.06] for control versus 6.87% [95% CI, 1.06–12.69] for ILR), respectively. The number needed to screen to avoid one stroke/SE after 6 years was 31 for participants with NT-proBNP >28 pmol/L corresponding to 233 pg/mL and 21 for participants with NT-proBNP >76 pmol/L corresponding to 633 pg/mL.

### AF Outcomes and OAC Initiation

**Figure 4.**
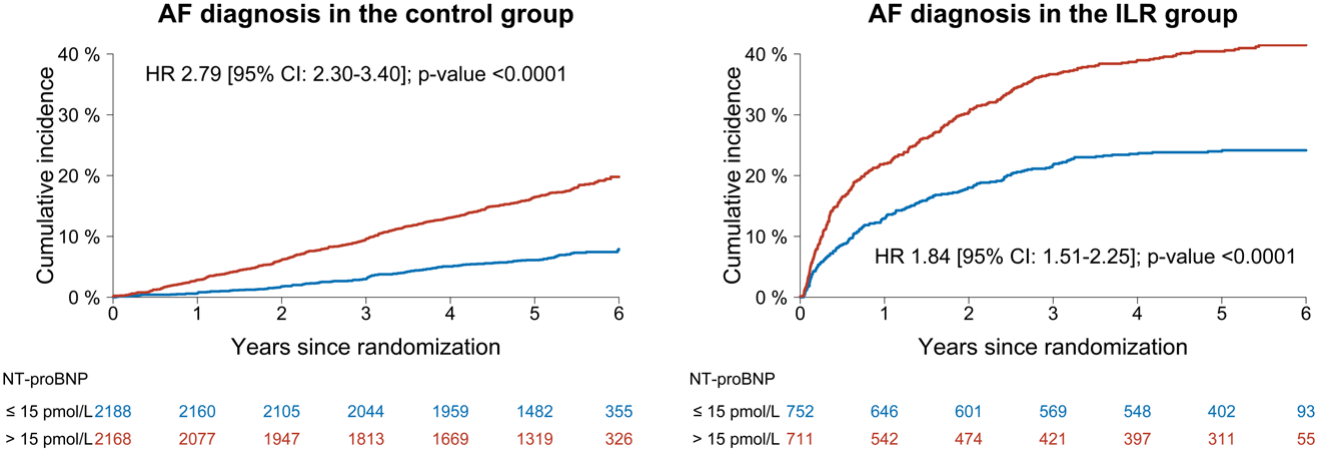
**Cumulative incidences of AF diagnosis according to NT-proBNP subgroups.** The absolute risk of atrial fibrillation (AF) diagnosis in the control and implantable loop recorder (ILR) groups according to NT-proBNP (N-terminal pro-B-type natriuretic peptide) subgroups. Cumulative incidences were plotted with the Aalen-Johansen estimator with death as competing risk. Hazard ratios (HRs) with 95% CI and *P* values were determined in cause-specific Cox models adjusted for sex, age, body mass index, weekly alcohol consumption, smoking pack-years, estimated glomerular filtration rate, total cholesterol concentration, hypertension, diabetes, previous stroke, heart failure, valvular heart disease, ischemic heart disease, and peripheral artery disease. The conversion factor for NT-proBNP expressed as pg/mL is 1 pg/mL=0.12 pmol/L. Hence, 15 pmol/L corresponds to 125 pg/mL.

In total, 1014 of 5819 participants (17.4%) were diagnosed with AF during follow-up: 544 of 4356 participants (12.5%) in the control group versus 470 of 1463 participants (32.1%) in the ILR group. Figure 4 depicts the cumulative incidences of AF diagnosis according to NT-proBNP subgroups. A baseline NT-proBNP level >15 pmol/L was associated with an increased risk of AF diagnosis in both the control group (HR 2.79 [95% CI, 2.30–3.40]) and the ILR group (HR 1.84 [95% CI, 1.51–2.25]). Similarly, during ILR monitoring with a median duration of 3.27 years (interquartile range, 3.06–3.45 years), participants with NT-proBNP >15 pmol/L were also more likely to experience ILR-detected AF episodes lasting ≥24 hours compared with those having lower levels (HR 2.45 [95% CI, 1.46–4.10]; Figure S4). For ILR screening compared with usual care (Table S4), the screening yield on AF diagnosis was significantly greater among participants with NT-proBNP ≤15 pmol/L (HR 4.06 [95% CI, 3.27–5.04]) than those with higher NT-proBNP levels (HR 2.89 [95% CI, 2.49–3.37]; *P*_interaction_=0.012).

Among the 1014 participants diagnosed with AF, 898 (88.6%) were started on OAC: 471 of 544 (86.6%) in the control group versus 427 of 470 (90.9%) in the ILR group. During follow-up, the event rate of OAC initiation was 1.63 (95% CI, 1.40–1.89) per 100 person-years in the control group and 5.37 (95% CI, 4.60–6.23) per 100 person-years in the ILR group among participants with NT-proBNP ≤15 pmol/L (HR 3.25 [95% CI, 2.64–4.00]). For participants with higher NT-proBNP levels, OAC initiation occurred at a rate of 3.92 (95% CI, 3.54–4.32) and 10.14 (95% CI, 8.96–11.45) per 100 person-years in the control group and ILR group, respectively, corresponding to an HR of 2.55 (95% CI, 2.18–2.98).

### Blood Pressure Management

Among the 5819 participants included, 5813 had available SBP measurement at baseline and 4788 at 3-year follow-up. For 3-year follow-up compared with baseline, SBP was reduced by 2.8 mm Hg (95% CI, 2.2–3.4) in the control group and 3.9 mm Hg (95% CI, 2.9–4.9) in the ILR group (*P*=0.049; Figure S5). When further stratified by NT-proBNP subgroups, the mean SBP reduction was 3.8 mm Hg (95% CI, 2.8–4.8) in the control group and 4.6 mm Hg (95% CI, 3.1–6.2) in the ILR group among participants with NT-proBNP >15 pmol/L (*P*=0.35). For those having lower NT-proBNP levels, SBP was reduced by 1.8 mm Hg (95% CI, 1.0–2.6) and 3.1 mm Hg (95% CI, 1.9–4.4) in the control group and ILR group, respectively (*P*=0.059).

## Discussion

This is the first study to assess the effects of AF screening on clinical outcomes according to NT-proBNP levels. In the present post hoc analysis of 5819 older individuals with additional stroke risk factors enrolled in a large AF screening trial, we reported the following major findings: (1) participants with higher levels of NT-proBNP were at higher risk of AF, stroke, and death; and (2) compared with usual care, ILR screening was associated with a significant reduction in stroke risk among participants with baseline NT-proBNP above median (>15 pmol/L) but not among those having lower levels.

While NT-proBNP is an established biomarker for the diagnosis and risk stratification of heart failure,^20,21^ its utility in the setting of AF is less clear. Mounting evidence from large epidemiological cohort studies has indicated NT-proBNP to be a strong, independent predictor of incident AF.^5–8^ Furthermore, the secondary analyses of the RE-LY (Randomized Evaluation of Long-Term Anticoagulation Therapy), ARISTOTLE (Apixaban for the Prevention of Stroke in Subjects With Atrial Fibrillation), and ENGAGE AF-TIMI 48 (Effective Anticoagulation With Factor Xa Next Generation in Atrial Fibrillation–Thrombolysis in Myocardial Infarction 48) trials consistently demonstrated a positive correlation between NT-proBNP and stroke risk in patients with AF.^9–11^ In line with those findings, our study confirms that participants with higher baseline levels of NT-proBNP were at increased risk of AF and stroke. This could potentially explain the favorable effects of ILR screening among participants with NT-proBNP >15 pmol/L and the absence of screening benefits for those with lower levels. In addition, the observed stroke risk reduction for ILR screening versus usual care in the participant group with NT-proBNP >15 pmol/L seems to be attributable mainly to OAC initiation upon AF detection rather than risk factor management, as no significant difference in SBP reduction during follow-up was found across the randomization groups among these participants. Another interesting observation from our study is the relatively smaller yield of screening among participants with NT-proBNP >15 pmol/L despite a greater stroke risk reduction for ILR versus control. Compared with usual care, ILR screening appeared to increase the rate of AF diagnosis by less than 3-fold among participants with NT-proBNP >15 pmol/L versus more than 4-fold among those with NT-proBNP ≤15 pmol/L. The discrepancy between screening yield and stroke prevention may well translate into NT-proBNP being able to identify those who are more likely to develop a clinically relevant AF phenotype. This notion is further supported by our finding of a higher risk of ILR-detected AF ≥24 hours among participants with NT-proBNP >15 pmol/L than those with lower levels, as a secondary analysis of ASSERT (Asymptomatic Atrial Fibrillation and Stroke Evaluation in Pacemaker Patients and the Atrial Fibrillation Reduction Atrial Pacing Trial) already revealed that the increased stroke risk of device-detected AF was related primarily to long-lasting episodes exceeding 24 hours.^22^ Still, it could also be speculated that high NT-proBNP merely acts as a risk marker of cardiovascular comorbidities and thereby mediates linkage between comorbidity burden and screening benefits. Countering this argument is a previous LOOP substudy that found that any effect of ILR screening on outcomes was upheld by the healthier participants, not those with established cardiovascular disease.^23^ In addition, previous research suggests that NT-proBNP may serve as an etiological indicator of ischemic stroke. A meta-analysis of 23 stroke studies pointed toward an association between elevated NT-proBNP and cardioembolic stroke,^24^ whereas a Japanese study of patients with AF with acute ischemic stroke reported a higher prevalence of coexisting small-vessel occlusion and/or large-artery atherosclerosis among those having lower levels of BNP (B-type natriuretic peptide).^25^ Indeed, increased wall tension in the atria has been proposed to stimulate atrial secretion of BNP and thus, elevated NT-proBNP may reflect atrial dysfunction.^26–28^ Given the documented correlation between atrial function and atrial thrombus formation,^29^ the observed differential effect of ILR screening according to NT-proBNP levels is therefore biologically plausible.

The latest guidelines from the European Society of Cardiology recommend considering systematic electrocardiogram screening in individuals aged ≥75 years or those at high stroke risk as assessed by the CHA_2_DS_2_-VASc score.^2^ The present study suggests that NT-proBNP may better identify the appropriate subpopulation who would potentially benefit from AF screening and subsequent management. Besides, when applying NT-proBNP >15 pmol/L as a cutoff, the number needed to screen to avoid one stroke after 6 years would be reduced from 62 in the entire population^4^ to 31. NT-proBNP is now also available as point-of-care test, with an expected decrease in cost over time. Hence, NT-proBNP may constitute an easily accessible and decisive tool for risk stratification with a potential to improve the cost-effectiveness of AF screening.

Of note, although the arbitrary cutoff of NT-proBNP >15 pmol/L appeared useful in the present exploratory analysis, the optimal cutoff to implement for AF screening in clinical practice has yet to be defined and validated. Indeed, according to our analyses of continuous NT-proBNP, a stroke risk reduction for ILR screening versus usual care already emerged at an NT-proBNP level from 9 pmol/L. However, it is worth mentioning that both European and American guidelines recommend using 125 pg/mL (ie, 15 pmol/L) as the threshold to rule out ventricular dysfunction in nonacute settings.^20,21^ The same cutoff has also been proposed for systematic AF screening in a subanalysis of the STROKESTOP (Systematic ECG Screening for Atrial Fibrillation Among 75 Year Old Subjects in the Region of Stockholm and Halland, Sweden) trial and is currently being tested in the ongoing STROKESTOP II trial.^13,14^ It is important to note that further studies are warranted to investigate potential harms from various thresholds for screening implementation such as anticoagulation-related bleeding risks and overdiagnosis of impaired ventricular function and the related downstream testing.

### Limitations

Several study limitations should be acknowledged. First, this was not a prespecified analysis of the LOOP study and the findings should be considered exploratory and hypothesis generating only. Second, echocardiographic data are lacking, although multivariable adjustments were performed as an attempt to accommodate confounders mediating the link between ventricular function and NT-proBNP. Third, due to the inclusion criteria of the LOOP study, our study population comprised solely individuals aged 70 to 90 years, which limits the generalizability of our results to other age groups. Finally, values for NT-proBNP expressed as pg/mL instead of pmol/L may differ slightly, depending on the number of significant figures in the conversion factor.

### Conclusions

In this exploratory post hoc analysis of a large, randomized trial of older individuals with additional stroke risk factors, higher NT-proBNP levels at baseline were associated with increased risks of AF diagnosis, stroke, and death. Compared with usual care, long-term continuous screening with ILR and subsequent OAC initiation upon AF detection were associated with a significant reduction in stroke risk among individuals with higher NT-proBNP levels but not among those with lower levels. These findings should be considered hypothesis generating, and future studies are warranted to assess the net benefits of AF screening according to NT-proBNP.

## Article Information

### Acknowledgments

The authors thank Dan Atar (Oslo University Hospital Ullevål, Norway) and Mårten Rosenqvist (Karolinska Institutet and Danderyd Hospital, Sweden) for their contributions to the international advisory committee of the LOOP study. They thank the research nurses and other colleagues in the Departments of Cardiology at Rigshospitalet, Bispebjerg, and Frederiksberg Hospital, Zealand University Hospital, and Odense University Hospital, who assisted with the conduct of the LOOP study.

### Sources of Funding

The LOOP study was supported by Innovation Fund Denmark (grant 12-1352259), the Research Foundation for the Capital Region of Denmark, the Danish Heart Foundation (grant 11-04-R83-A3363-22625), Aalborg University Talent Management Program, Arvid Nilssons Fond, Skibsreder Per Henriksen, R og Hustrus Fond, the European Union Horizon 2020 program (grant 847770 to the AFFECT-EU Consortium), Læge Sophus Carl Emil Friis og hustru Olga Doris Friis’ Legat, and an unrestricted grant from Medtronic. Dr Xing’s employment is funded by the AFFECT-EU Consortium and thereby the European Union Horizon 2020 program (grant 847770).

### Disclosures

Dr Svendsen reports being a member of Medtronic advisory boards and having received speaker honoraria and research grants from Medtronic in relation to this work and outside this work. Dr Diederichsen reports being a part-time employee of VitalBeats and an advisor at Bristol-Myers Squibb/Pfizer, not related to this work. Dr Krieger reports being a Medtronic focus group member. Dr Frikke-Schmidt reports speaker honorarium and consultant fees from Novo Nordisk. Dr Brandes reports research grants from the Region of Zealand, the Canadian Institutes of Health Research, the Danish Heart Foundation, and Theravance, as well as speaker honoraria from Boehringer Ingelheim and Bristol-Myers Squibb, not related to this work. Dr Køber reports speaker honoraria from Novo Nordisk, AstraZeneca, Novartis, and Boehringer, not related to this work. The other authors report no conflicts.

### Supplemental Material

Tables S1–S4

Figures S1–S5

## Supplementary Material
