# Supplementary material for: Effects of Atrial Fibrillation Screening According to N-Terminal Pro-B-Type Natriuretic Peptide: A Secondary Analysis of the Randomized LOOP Study
Source: Circulation. 2023 Apr 15;147(24):1788–97. doi: 10.1161/CIRCULATIONAHA.123.064361 (PMC10249603; doi:10.1161/CIRCULATIONAHA.123.064361)
Supplement: Supplementary file 1 [file cir-147-1788-s001.pdf]

SUPPLEMENTAL MAETRIAL

Supplemental tables

Table S1: Conversion of NT-proBNP from pmol/L to pg/mL

Table S2: Akaike Information Criterion score for different models of the relationships between continuous NT-proBNP levels and screening effects on primary and secondary outcomes

Table S3: Crude event rates and hazard ratios of primary and secondary outcomes in the entire cohort according to NT-proBNP subgroups

Table S4: Crude event rates and hazard ratios of AF diagnosis for ILR versus Control according to NT-proBNP subgroups

Supplemental figures

Figure S1: The distributions of NT-proBNP in the study population

Figure S2: The associations of primary and secondary outcomes with NT-proBNP as continuous variable

Figure S3: Cumulative incidences of primary and secondary outcomes according to randomization assignment stratified by NT-proBNP subgroups

Figure S4: Cumulative incidences of ILR-detected AF episodes ≥24 hours according to NT-proBNP subgroups

Figure S5: Changes in systolic blood pressure at 3-year follow-up

Table S1: Conversion of NT-proBNP from pmol/L to pg/mL

**NT-proBNP [pmol/L]**	9	15	28	76
**NT-proBNP [pg/mL]**	75	125	233	633

The conversion factor for NT-proBNP expressed as pg/mL is 1 pg/mL = 0.12 pmol/L.

Abbreviations: NT-proBNP, N-terminal pro-B-type natriuretic peptide.

Table S2: Akaike Information Criterion score for different models of the relationships between continuous NT-proBNP levels and screening effects on primary and secondary outcomes

	Linear relationship with NT-proBNP on the original scale	Linear relationship with log-transformed NT-proBNP	Restricted cubic spline model
**Stroke or SE**	5233.03	**5219.72**	5226.21
**Stroke, SE or cardiovascular death**	7863.97	**7815.90**	7821.99
**All-cause death**	11,166.36	**11,069.53**	11,076.62

The restricted cubic spline model assessed NT-proBNP as a continuous variable, with knots located at the 5th. 35th, 65th, and 95th percentiles.

Abbreviations: NT-proBNP, N-terminal pro-B-type natriuretic peptide; SE, systemic embolism.

Table S3: Crude event rates and hazard ratios of primary and secondary outcomes in the entire cohort according to NT-proBNP subgroups

Endpoints	NT-proBNP	Events	Participants	Event rates	Hazard ratios
**Stroke or SE**	≤15 pmol/L	135	2940	0.89 [0.74-1.05]	Reference
	>15 pmol/L	175	2879	1.21[1.04-1.40]	1.21 [0.96-1.54]
**Stroke, SE, or cardiovascular death**	≤15 pmol/L	172	2940	1.13[0.97-1.31]	Reference
	>15 pmol/L	298	2879	2.06 [1.83-2.31]	**1.60 [1.32-1.95]**
**All-cause death**	≤15 pmol/L	214	2940	1.38 [1.20-1.58]	Reference
	>15 pmol/L	455	2879	3.07 [2.80-3.37]	**1.91 [1.61-2.26]**

Crude event rates are presented as event number per 100 person-years. Hazard ratios are presented with 95% confidence intervals and were estimated in cause-specific Cox regression models adjusted for sex, age, body mass index, weekly alcohol consumption, smoking pack years, estimated glomerular filtration rate, total cholesterol concentration, hypertension, diabetes, prior stroke, heart failure, valvular heart disease, ischemic heart disease, and peripheral artery disease.

The conversion factor for NT-proBNP expressed as pg/mL is 1 pg/mL = 0.12 pmol/L. Hence, 15 pmol/L corresponds to 125 pg/mL.

Abbreviations: NT-proBNP, N-terminal pro-B-type natriuretic peptide; SE, systemic embolism.

Table S4: Crude event rates and hazard ratios of AF diagnosis for ILR versus Control according to NT-proBNP subgroups

NT-proBNP Groups	Control group		ILR group		Hazard ratios	$p_{interaction}$
	Event / participant	Event rates	Event / participant	Event rates		
≤15 pmol/L	152 / 2188	1.35 [1.15-1.59]	181 / 752	5.60 [4.82-6.48]	**4.06 [3.27-5.04]**	**0.012**
>15 pmol/L	392 / 2168	3.83 [3.46-4.23]	289 / 711	11.42 [10.14-12.82]	**2.89 [2.49-3.37]**	

Crude event rates are presented as event number per 100 person-years. Hazard ratios and p-value for interaction were estimated in cause-specific Cox regression models.

The conversion factor for NT-proBNP expressed as pg/mL is 1 pg/mL = 0.12 pmol/L. Hence, 15 pmol/L corresponds to 125 pg/mL.

Abbreviations: AF, atrial fibrillation; ILR, implantable loop recorder; NT-proBNP, N-terminal pro-B-type natriuretic peptide.

<u>Figure S1</u>: The distributions of NT-proBNP in the study population

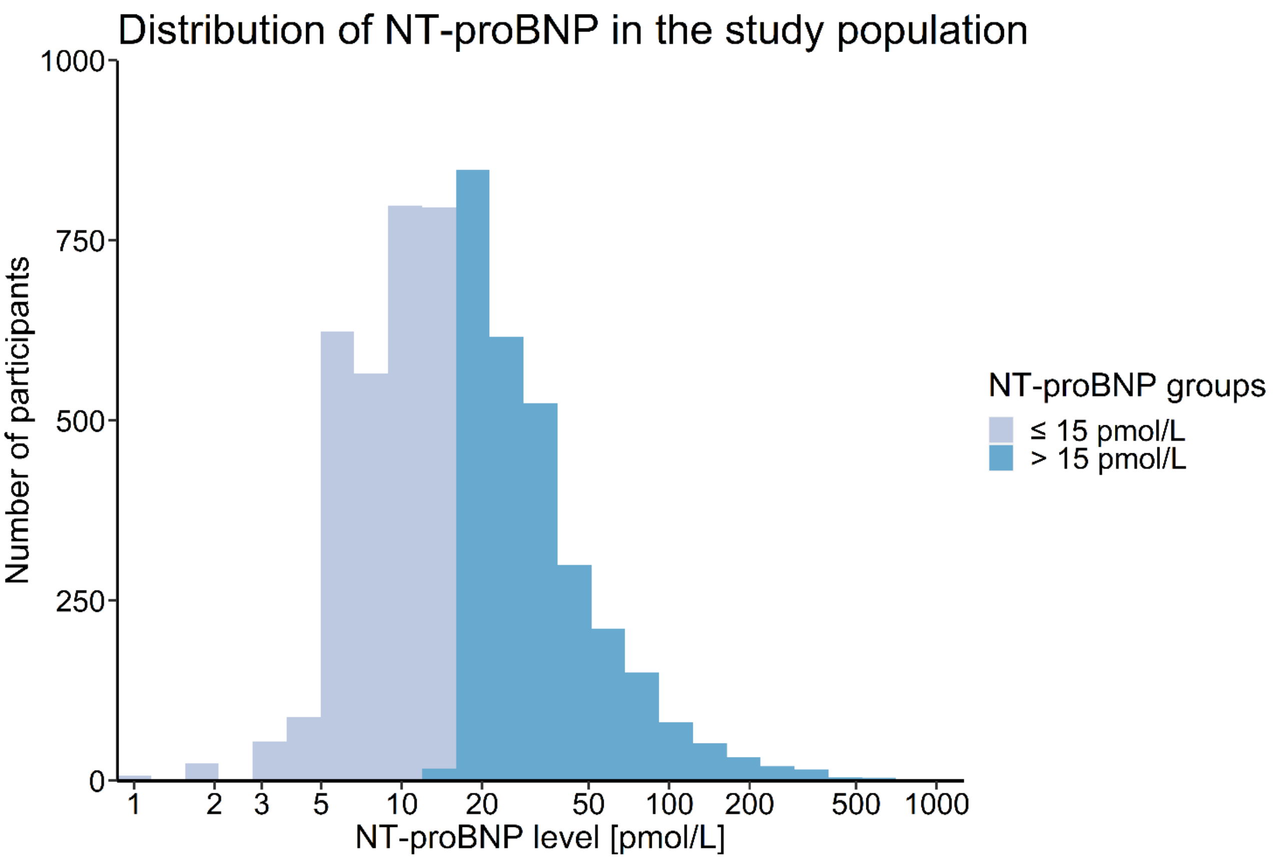

The figure shows the distribution of NT-proBNP among our study participants, with the x-axis logarithmically transformed. Different colors represent the dichotomized intervals of NT-proBNP.

The conversion factor for NT-proBNP expressed as pg/mL is 1 pg/mL = 0.12 pmol/L. Hence, 15 pmol/L corresponds to 125 pg/mL.

Abbreviation: NT-proBNP, N-terminal pro-B-type natriuretic peptide.

<u>Figure S2</u>: The associations of primary and secondary outcomes with NT-proBNP as continuous variable

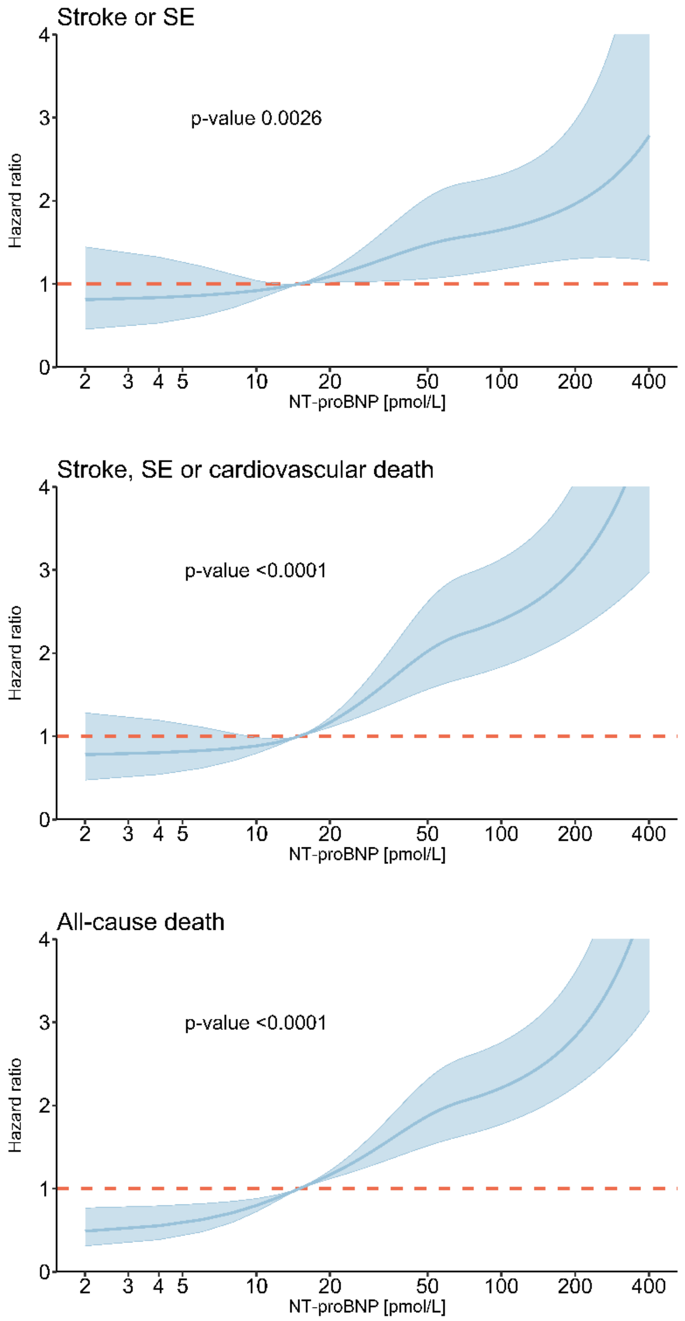

The figure shows the risks of stroke/SE, stroke/SE/cardiovascular death, and all-cause death in the entire study cohort according to NT-proBNP as a continuous variable. Hazard ratios were estimates with the median NT-proBNP value (15 pmol/L) as reference, in cause-specific Cox models adjusted for sex, age, body mass index, weekly alcohol consumption, smoking pack years, estimated glomerular filtration rate, total cholesterol concentration, hypertension, diabetes, prior stroke, heart failure, valvular heart disease, ischemic heart disease, and peripheral artery disease. The colored areas represent 95% confidence intervals.

The conversion factor for NT-proBNP expressed as pg/mL is 1 pg/mL = 0.12 pmol/L. Hence, 15 pmol/L corresponds to 125 pg/mL.

Abbreviations: NT-proBNP, N-terminal pro-B-type natriuretic peptide; SE, systemic embolism.

<u>Figure S3</u>: Cumulative incidences of primary and secondary outcomes according to randomization assignment stratified by NT-proBNP subgroups

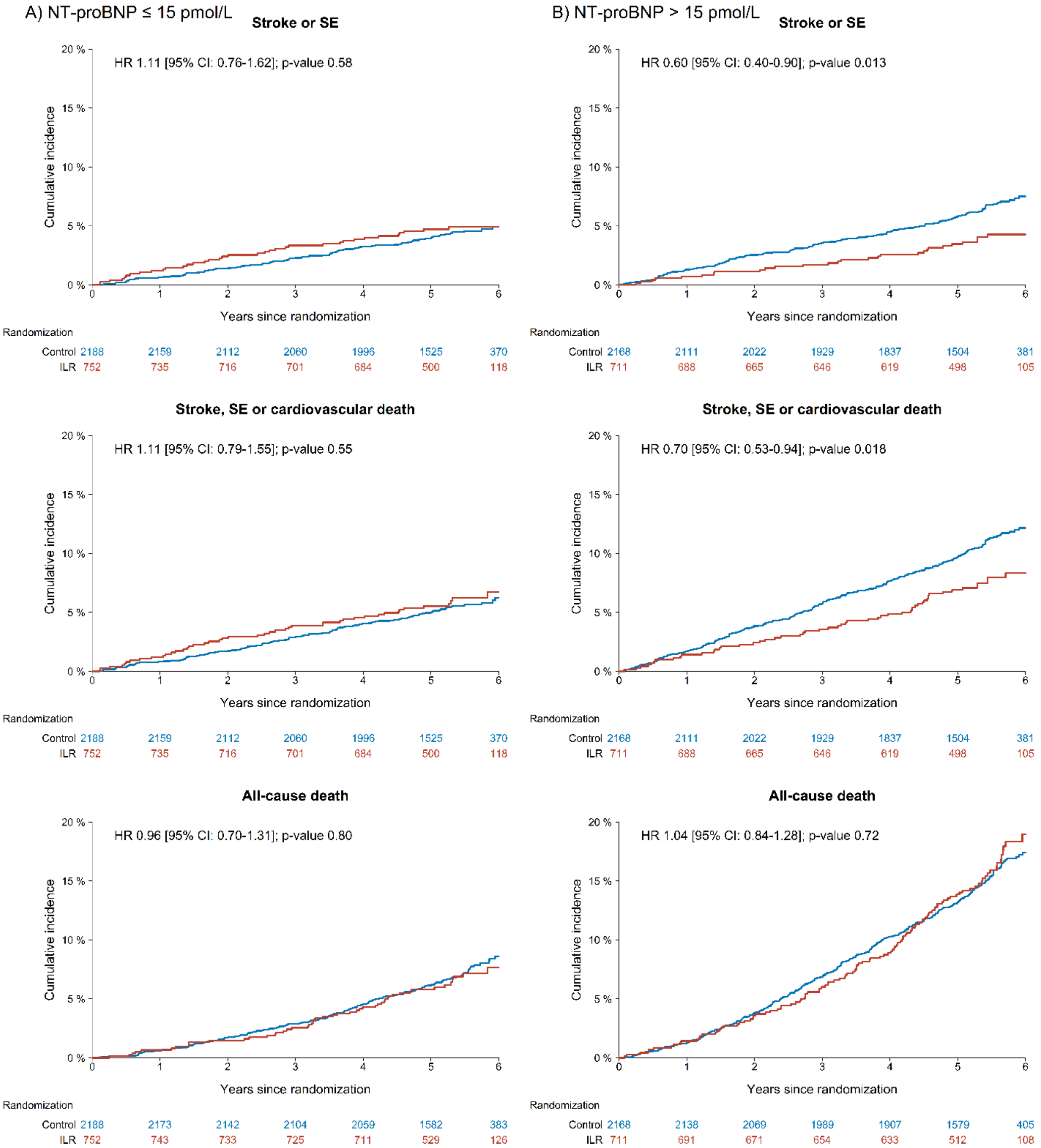

The figure shows the absolute risks of stroke/SE, stroke/SE/cardiovascular death, and all-cause death according to randomization assignment among participants with baseline NT-proBNP ≤15 pmol/L (**panel A**) and >15 pmol/L (**panel B**). Cumulative incidences were plotted using the Kaplan-Meier estimator for all-cause death and the Aalen-Johansen estimator for other outcomes with death as competing risk. HRs and p-values were estimated in cause-specific Cox regression models.

The conversion factor for NT-proBNP expressed as pg/mL is 1 pg/mL = 0.12 pmol/L. Hence, 15 pmol/L corresponds to 125 pg/mL.

Abbreviations: CI, confidence interval; HR, hazard ratio; ILR, implantable loop recorder; NT-proBNP, N-terminal pro-B-type natriuretic peptide; SE, systemic embolism.

<u>Figure S4</u>: Cumulative incidences of ILR-detected AF episodes ≥24 hours according to NT-proBNP subgroups

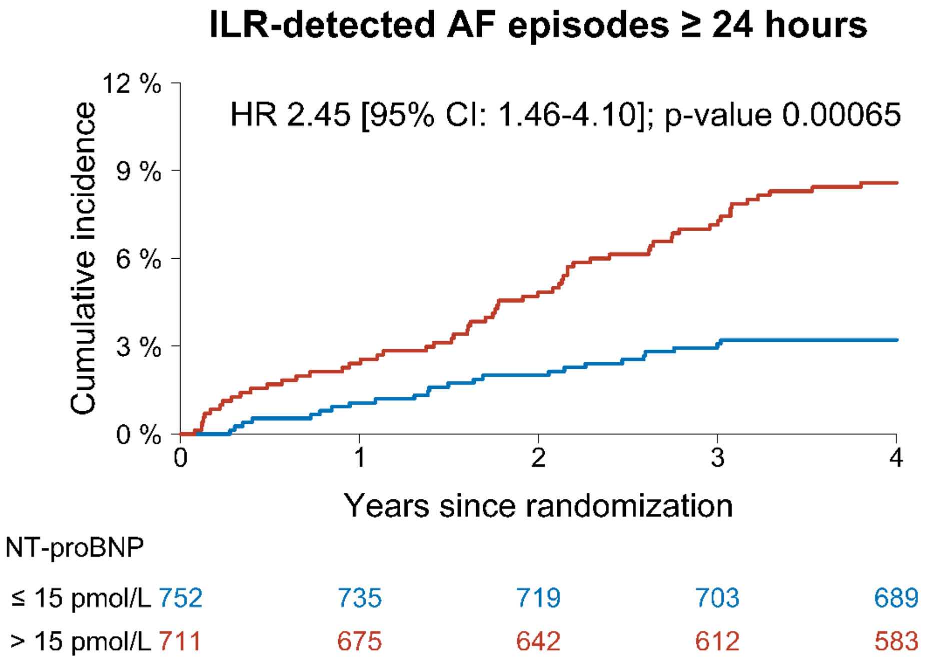

The figure shows the absolute risk of ILR-detected AF episodes ≥24 hours in the ILR group, according to NT-proBNP subgroups. The curves are truncated after four years, which was the maximum duration of ILR monitoring in the LOOP Study. Cumulative incidences were plotted using the Aalen-Johansen estimator with death as competing risk. HRs and p-values were determined in cause-specific Cox models adjusted for sex, age, body mass index, weekly alcohol consumption, smoking pack years, estimated glomerular filtration rate, total cholesterol concentration, hypertension, diabetes, prior stroke, heart failure, valvular heart disease, ischemic heart disease, and peripheral artery disease.

The conversion factor for NT-proBNP expressed as pg/mL is 1 pg/mL = 0.12 pmol/L. Hence, 15 pmol/L corresponds to 125 pg/mL.

Abbreviations: AF, atrial fibrillation; Cl, confidence interval; HR, hazard ratio; ILR, implantable loop recorder; NT-proBNP, N-terminal pro-B-type natriuretic peptide.

<u>Figure S5</u>: Changes in systolic blood pressure at 3-year follow-up

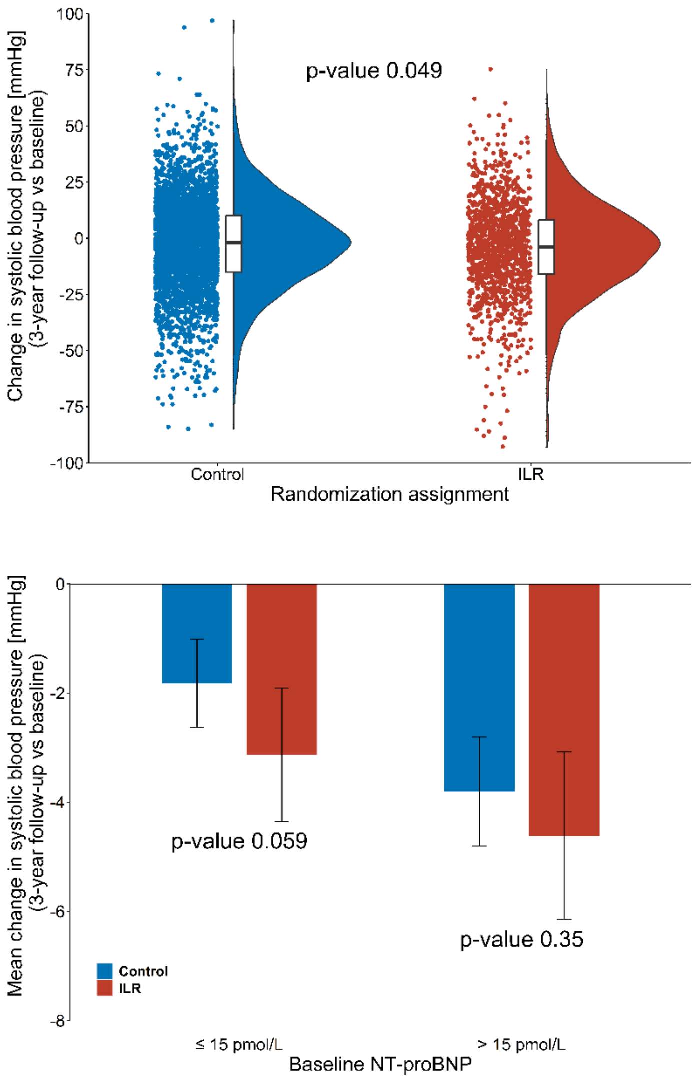

The figure shows the distributions of 3-year changes in systolic blood pressure according to randomization assignment (**upper panel**) and mean changes in systolic blood pressure in participants with baseline NT-proBNP levels ≤15 and >15 pmol/L (**lower panel**). Error bars represent 95% confidence intervals of mean blood pressure changes.

The conversion factor for NT-proBNP expressed as pg/mL is 1 pg/mL = 0.12 pmol/L. Hence, 15 pmol/L corresponds to 125 pg/mL.

Abbreviations: ILR, implantable loop recorder; NT-proBNP, N-terminal pro-B-type natriuretic peptide.
